# Subepicardial Cardiomyopathy: A Disease Underlying J-Wave Syndromes and Idiopathic Ventricular Fibrillation

**DOI:** 10.1161/CIRCULATIONAHA.122.061924

**Published:** 2023-05-23

**Authors:** Chris Miles, Bastiaan J. Boukens, Chiara Scrocco, Arthur A.M. Wilde, Koonlawee Nademanee, Michel Haissaguerre, Ruben Coronel, Elijah R. Behr

**Affiliations:** 1 Cardiovascular Clinical Academic Group, St. George’s University Hospitals’ NHS Foundation Trust and Molecular and Clinical Sciences Institute, St. George’s, University of London, UK (C.M., C.S., E.R.B.).; 2 Department of Medical Biology, University of Amsterdam, the Netherlands (B.J.B.).; 3 University of Maastricht, Cardiovascular Research Institute Maastricht, Maastricht University Medical Center, the Netherlands (B.J.B.).; 4 Amsterdam UMC, University of Amsterdam, Department of Cardiology, the Netherlands (A.A.M.W.).; 5 Amsterdam Cardiovascular Sciences, Heart Failure and Arrhythmias, the Netherlands (A.A.M.W.).; 6 European Reference Network for rare, low-prevalence, and complex diseases of the heart: ERN GUARD-Heart (A.A.M.W., M.H.).; 7 Center of Excellence in Arrhythmia Research Chulalongkorn University, Department of Medicine, Chulalongkorn University, Thailand (K.N.).; 8 Pacific Rim Electrophysiology Research Institute, Bumrungrad Hospital, Bangkok, Thailand (K.N.).; 9 Institut Hospitalo-Universitaire Liryc, Electrophysiology and Heart Modeling Institute, Pessac, France (M.H.).; 10 Department of Electrophysiology and Cardiac Stimulation, Centre Hospitalier Universitaire de Bordeaux, France (M.H.).; 11 Department of Experimental Cardiology, Amsterdam University Medical Centers, Cardiovascular Science, the Netherlands (R.C.).; 12 Mayo Clinic Healthcare, London, UK (E.R.B.).

**Keywords:** arrhythmogenic cardiomyopathies, Brugada syndrome, ventricular fibrillation

## Abstract

Brugada syndrome (BrS), early repolarization syndrome (ERS), and idiopathic ventricular fibrillation (iVF) have long been considered primary electrical disorders associated with malignant ventricular arrhythmia and sudden cardiac death. However, recent studies have revealed the presence of subtle microstructural abnormalities of the extracellular matrix in some cases of BrS, ERS, and iVF, particularly within right ventricular subepicardial myocardium. Substrate-based ablation within this region has been shown to ameliorate the electrocardiographic phenotype and to reduce arrhythmia frequency in BrS. Patients with ERS and iVF may also exhibit low-voltage and fractionated electrograms in the ventricular subepicardial myocardium, which can be treated with ablation. A significant proportion of patients with BrS and ERS, as well as some iVF survivors, harbor pathogenic variants in the voltage-gated sodium channel gene, *SCN5A*, but the majority of genetic susceptibility of these disorders is likely to be polygenic. Here, we postulate that BrS, ERS, and iVF may form part of a spectrum of subtle subepicardial cardiomyopathy. We propose that impaired sodium current, along with genetic and environmental susceptibility, precipitates a reduction in epicardial conduction reserve, facilitating current-to-load mismatch at sites of structural discontinuity, giving rise to electrocardiographic changes and the arrhythmogenic substrate.

Some arrhythmia syndromes appear to occur in the absence of overt structural abnormalities. The long QT syndrome is one such example.^1^ The now well-defined pathophysiological basis of long QT syndrome and associated arrhythmias in the absence of structural myocardial abnormalities characterizes the syndrome as a dominant (if not 100% genetic) ion channel disease, a channelopathy. Various other channelopathies have been described, including Brugada syndrome (BrS), catecholaminergic polymorphic ventricular tachycardia, and short QT syndrome. BrS, a leading cause of autopsy-negative sudden death,^2^ is defined by coved J-point (ST-segment) elevation in the right precordial leads in association with ventricular fibrillation (VF) in the absence of structural abnormalities. J-point elevation is also a requisite feature of the early repolarization syndrome (ERS), which refers to the finding of early repolarization pattern in patients with idiopathic VF (iVF). BrS and ERS therefore constitute a continuous spectrum of J-wave phenotypic expression in the ECG, and thus have been designated J-wave syndromes.^3^ Other clinical entities can mimic the electrocardiographic pattern observed in BrS, but are etiologically distinct and elicited by other factors, such as myocardial ischemia, metabolic abnormalities, or mechanical compression.^4^ Early repolarization pattern is also more commonly observed in competitive athletes compared with the general population.^5^ In the absence of an overt electrical or structural phenotype, iVF exists as a diagnosis of exclusion, referring to the occurrence of VF without a pathophysiological explanation.^6^

Because the 3 disorders, in at least some patients, share the presence of subtle changes in the extracellular matrix, a common pathophysiological basis for BrS, ERS, and iVF appears plausible (Figure 1). The presence of a vulnerable electrophysiological substrate, in conjunction with triggers commonly arising from the Purkinje system or right ventricular (RV) outflow tract (RVOT), likely plays an important role in arrhythmogenesis, particularly when combined with genetic and environmental modifiers. This is consistent with our view that syndromic descriptions of BrS and ERS point to a different region of the heart and to a different severity of the microstructural changes, whereas iVF may be associated with abnormalities in various regions. In contrast to primary cardiomyopathic disorders, in which the heart muscle appears both structurally and functionally abnormal, alterations in ion channel interfaces and protein architecture have led some to recognize cardiac channelopathies as a subgroup of primary cardiomyopathies rather than purely electrical diseases.^7^ We therefore propose that most patients with BrS, ERS, or iVF have a common subepicardial cardiomyopathy based on nontransmural, subtle microstructural changes present within the subepicardial myocardium.^8^ Whether these microstructural changes are also present in patients with other primary electrical diseases is unclear, although a recent study by Pappone et al^9^ suggests that, in patients with long QT syndrome, cardiac structural abnormalities may be present as well. Here, we discuss the replacement of the syndromic descriptions of BrS, ERS, and iVF with a common unifying pathophysiological definition.

**Figure 1.**
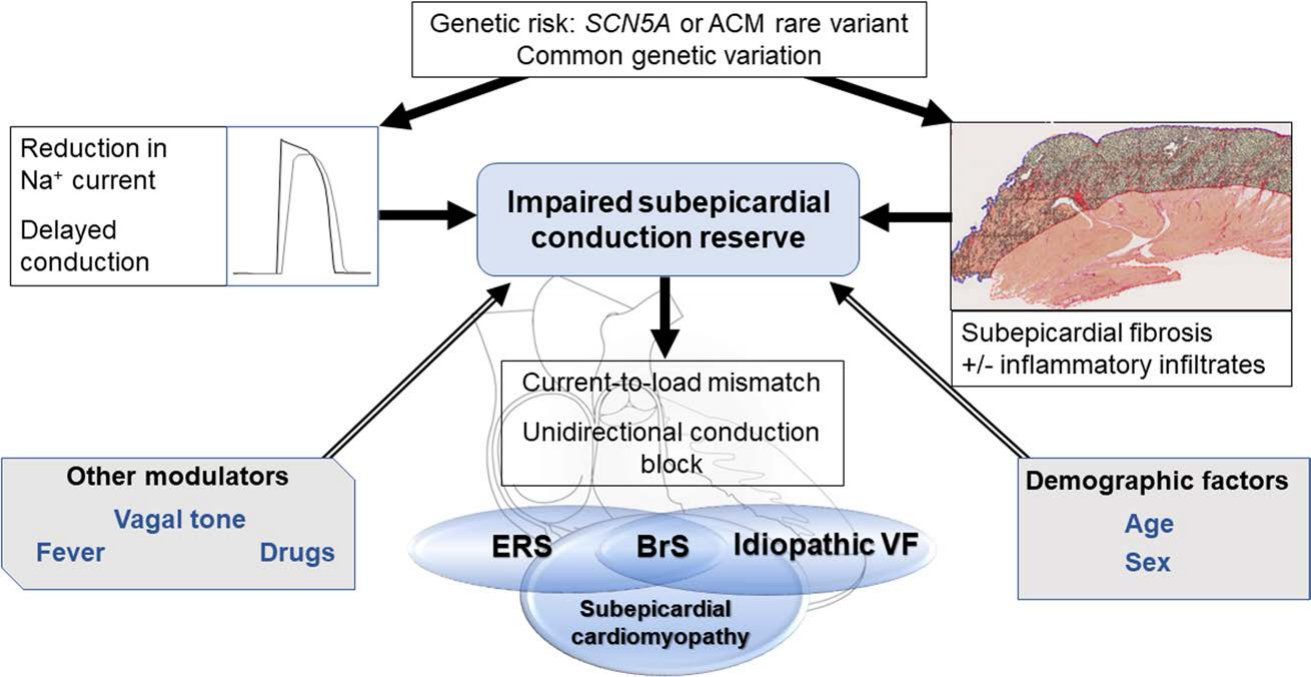
**Proposed mechanisms and modulating factors underlying arrhythmogenesis in the subepicardial cardiomyopathy, giving rise to BrS, ERS, and idiopathic VF.** ACM indicates arrhythmogenic cardiomyopathy; BrS, Brugada syndrome; ERS, early repolarization syndrome; and VF, ventricular fibrillation.

## Evidence of Structural Disease in These Phenotypes

The presence of an apparently abnormal myocardial substrate in BrS has been widely reported in the literature, but the histological descriptions vary. Cardiomyopathic changes, including myocardial fibrofatty replacement of the RV free wall or the presence of inflammatory infiltrates, have been described in a series of studies.^10–13^ Microstructural abnormalities have been reported in an earlier case series of 6 individuals with apparent iVF, one of whom fulfilled contemporary electrocardiographic criteria for BrS.^14^ A recent study by Miles et al^15^ reported pathological and clinical characteristics in a group of 28 decedents with BrS compared with control subjects without cardiac death. Quantitative analysis of cardiac tissue components demonstrated a 45% increase in collagen content among the BrS group compared with control subjects. Increases in collagen were observed across all sampled regions of RV and left ventricular myocardium, with the highest collagen proportions within the subepicardium of the RVOT. These findings suggest that fibrosis predominates in RV subepicardial myocardium but also appears to represent an adverse remodeling process in both ventricles. However, the syndromic description of BrS classifies patients as having RVOT disease, failing to recognize its true extent.^16^

Although BrS and ERS are often considered distinct arrhythmia syndromes, overlapping clinical and pathophysiological features are increasingly recognized. It has been proposed that the electrocardiographic J wave, previously characterized in experimental studies by accentuation of the notch in the subepicardial action potential (caused by the transient outward potassium current [*I*_to_]), can also be caused by or related to activation delay,^17,18^ underpinning a spectrum of BrS and ERS disease phenotypes associated with malignant ventricular arrhythmias and sudden cardiac death.^3^ The conventional view of ERS as an exclusive disorder of enhanced local early repolarization in the absence of apparent structural cardiac abnormalities has also been challenged.^19,20^ Boukens et al^20^ recently documented the presence of fibrosis within regions of inferior RV myocardium colocalizing with electrophysiological J waves observed during epicardial unipolar mapping. Here, transmural myocardial biopsies were obtained from a patient with ERS with recurrent VF undergoing epicardial mapping and ablation. High-resolution activation mapping identified the latest moment of electrical activation within the inferior RV free wall, in which high-amplitude local J waves were present on unipolar recordings occurring after the moment of slurring/notching in the QRS complex of the ECG. Extensive subepicardial fibrosis was observed histologically, along with fragments of surviving myocardium.

In patients with iVF, microstructural changes with potentials similar to those observed in BrS can also occur. More than two-thirds of 50 patients with iVF who underwent comprehensive exclusion of underlying cardiac causes showed evidence of low-amplitude and fractionated electrograms detected during electrophysiological catheter mapping procedures, indicative of abnormal conduction and arrhythmogenicity.^21^ Abnormal conduction was found predominantly in RV (65%) subepicardial myocardium, whereas Purkinje premature ventricular contractions were the dominant cause in patients with iVF without conduction alterations. Concordant with biventricular fibrotic involvement in BrS,^15^ microstructural alterations of the left ventricular myocardium with or without the RV myocardium were identified in a significant minority (35%). Localized abnormal electrograms within both ventricles were also commonly reported in a series of patients with iVF subjected to endocardial and epicardial mapping.^22^ Here, Haïssaguerre et al^22^ identified sites of abnormal ECGs among 15 of 24 patients with iVF. Furthermore, abnormal areas were found to colocate with VF drivers; clinical recurrences were reduced after substrate-based ablation. The presence of myocardial fibrosis and fatty infiltration in cardiac tissue has also been associated with the distribution of J waves on the 12-lead ECG.^23^

## A Common Underlying Pathophysiology?

The underlying pathophysiology of BrS has been a matter of much debate.^24^ The 2 main electrophysiological hypotheses are the repolarization and depolarization theories.^25^ The repolarization theory is based on experiments in perfused canine RV wedge preparations and refers to transmural dispersion of repolarization in the absence of structural abnormalities.^26^ According to this hypothesis, the notch of the action potential is accentuated due to reduction of net inward Na^+^ current (NaV1.5), along with nonuniform increases in the *I*_to_ within the subepicardial myocardium. In the depolarization theory, ST-segment elevation observed in the right precordial leads is explained by severely compromised conduction, including slow or asynchronous conduction, localized block, and absence of activation within the RVOT, creating a large potential difference with respect to the body of the RV.^27–30^ Reduction of sodium current by sodium channel blockade, the presence of a *SCN5A* pathogenic variant, high-rate pacing, or extrasystoles can unmask the substrate.^27,29^ Experimental models also provide a mechanistic basis for the association between fibrosis and BrS. For example, a study using a haploinsufficient *SCN5A*^+/−^ mouse model demonstrated fibrotic changes within both ventricles; epicardial activation analysis also showed increased late conducting components.^31^ Conduction deficits and myocardial fibrosis have been elegantly described in a porcine model of *SCN5A* deficiency, underscoring the pleotropic nature of sodium channel disease.^32^

It has been proposed that a reduction of sodium current is caused by current-to-load mismatch and localized conduction block, resulting in excitation failure within fibrotic myocardium in the RV epicardium.^28^ In a porcine model and in computer simulations, Hoogendijk et al^29^ showed that localized excitation failure by current-to-load mismatch can cause ST-segment elevation modulated by the balance of sodium current, I_to_, and calcium current. In the presence of structural discontinuities, a decrease in depolarizing (or an increase in the repolarizing) current may result in unidirectional conduction block. These findings are concordant with clinical data demonstrating excitation failure and localized RV epicardial activation delay in BrS myocardium.^33,34^

The most compelling data in favor of the depolarization hypothesis were put forward by Nademanee et al,^30^ who studied 9 patients with BrS with recurrent VF and frequent implantable cardioverter defibrillator discharges. Electroanatomic mapping showed low-voltage fragmented electrograms of prolonged duration over the epicardial aspect of the RVOT. Catheter ablation resulted in normalization of the type 1 Brugada electrocardiographic pattern, and no further arrhythmia was inducible, a phenomenon also observed by others.^35^ In our view, these findings suggested that localized J-point and ST-segment elevation is a consequence of delayed depolarization of the RVOT with current-to-load mismatch at areas of cardiac tissue discontinuity. This was made plausible by recording of delayed monophasic unipolar electrograms after sodium channel blocker administration in patients with BrS with or without early repolarization pattern.^36^ A monophasic morphology of a unipolar electrogram is commonly accepted as a sign of absence of local activation.^37,38^ These monophasic potentials are visible in lead V_1_ as a J-point elevation.^39^

Similar observations have been made in patients with ERS.^19,20^ In an electroanatomic study of 58 patients with inferolateral J waves/ERS, 2 distinct electrical subtypes were identified. The majority was made up of those with depolarization abnormalities located predominantly at the inferior part of RV epicardium. A smaller group included individuals with no apparent depolarization abnormality but early repolarization unipolar signals (pure ERS) in which Purkinje-related VF triggers likely predominate.^19^ Mechanisms underlying iVF often relate to the presence of premature ventricular contraction triggers arising from the distal Purkinje system, and this classification should be considered after careful exclusion of covert structural or molecular cardiac causes.

Others have questioned the role of delayed conduction in BrS. The canine wedge model demonstrated fractionated electrograms and late potentials as a consequence of perturbations in epicardial repolarization (reactivation of calcium current) and action potential duration,^40^ although these electrograms show different characteristics in timing and continuity compared with those in human patients with BrS.^39^ Radiofrequency ablation of myocardium showing fractionated potentials mitigated the BrS electrocardiographic phenotype.^41^ Furthermore, important differences have also been described in the electrocardiographic response of BrS and ERS to sodium channel blockade,^42^ which appears to accentuate J-wave amplitude in BrS while causing a reduction in ERS. Although this suggests that distinct mechanisms may underlie both conditions, computer simulation data indicate that differences in J-wave manifestation occur due to regional patterns of delayed activation and reduction in sodium current. Additional conduction slowing in the entire heart (eg, by sodium channel blockade) may attenuate J waves and J-point elevations on the ECG because of masking due to global QRS widening.^18^ In humans with BrS, the presence of late potentials on the signal-averaged ECG has been associated with a positive response to the sodium channel blocker provocation test.^43^

## Underlying Genetic Causes

Since the landmark discovery of pathogenic variants in the first gene linked to BrS,^44^ *SCN5A* remains the only gene consistently associated with the clinical phenotype. To date, >300 mutations in *SCN5A* have been associated with BrS that underlie ≈20% of patients meeting diagnostic criteria.^45^ Pathogenic *SCN5A* variants in BrS cause loss of function due to reduction in the amplitude of the sodium channel current by reduced expression or altered voltage-gating properties. *SCN5A* variants have been described in various other cardiac pathologies, including long QT syndrome, premature cardiac conduction defect, and dilated cardiomyopathy. However, it should be noted that not all *SCN5A* variants are pathogenic, according to the Koch or Bradford Hill criteria.^46,47^ In fact, Probst et al^48^ found that within families with hereditary BrS and a pathogenic *SCN5A* variant, the genetic variant can be absent in symptomatic patients who comply with the syndromic criteria. Furthermore, given the relatively modest monogenic contribution of *SCN5A* to the BrS phenotype, it is clear that inheritance patterns are more complex than previously thought.^49^

Bezzina and et al^50^ provided initial support for this idea through a genome-wide association study exploring the role of common genetic variation in BrS. They identified 3 loci associated with BrS: rs10428132 and rs11708996, both at *SCN5A*/*SCN10A*, and rs9388451 near *HEY2*. These common variants were thought to account for ≈7% of variance in BrS susceptibility. Furthermore, disease risk increased consistently with increasing numbers of carried risk alleles. A follow-up study suggested that the weighted contribution of these variants may allow an individualized approach to diagnosis along with established clinical factors.^51^ A strong polygenic susceptibility was underscored by a further, much larger genome-wide association study implicating 21 common variants at 12 loci in BrS.^52^

The presence of *SCN5A* variants has also been reported in ERS, albeit at a lower diagnostic yield.^53,54^ In a study of 262 probands with BrS and 104 with ERS, Zhang et al^54^ identified a 10% yield of pathogenic *SCN5A* variants in the ERS group compared with 23% for BrS. This is further supported by patients with ERS undergoing ablation being more likely to harbor an *SCN5A* variant.^19^ This suggests that overlapping genetic features may underlie a significant minority of J-wave syndromes, ultimately contributing to reduced conduction reserve within RV epicardium.^55^ Furthermore, previous studies have shown that NaV1.5 may also have a role in the maintenance of normal cardiac structural integrity. Loss of NaV1.5 in heterozygous *SCN5A*^+/−^ murine models has resulted in conduction defects, in keeping with premature cardiac conduction defect, and the occurrence of age-dependent fibrotic cardiac remodeling, which appears to be triggered by activation of a transforming growth factor-β signaling pathway.^31,56,57^

## Evidence for Genetic Overlap With Arrhythmogenic Cardiomyopathy

Genetic variants in the desmosomal gene *PKP2* have been associated with clinically affected patients with BrS, and, conversely, *SCN5A* has been implicated in the pathogenesis of arrhythmogenic cardiomyopathy (ACM).^58,59^ The majority of annotated ACM pathogenic *PKP2* variants are radical alternations (frameshift or nonsense mutations), but nonsynonymous variants have also been associated with additional cardiac phenotypes such as BrS.^60^ This suggests a pleotropic role of the plakophilin-2 protein, which may have additional functions besides linking cadherins to intermediate filaments in the cytoskeleton.^61^ These findings are emphasized by experimental models detailing a molecular interaction between desmosomal proteins and the sodium channel, suggesting that both disease states may exist on a continuum, manifesting variable degrees of electrical and structural dysfunction.^62,63^ For example, biochemical, patch clamp, and optical mapping experiments have reported important associations between plakophilin-2 and NaV1.5 at a cellular level while also demonstrating adverse effects of *PKP2* knockdown on sodium current function.^64^ Similarly, *PKP2* variants were functionally detrimental to sodium channel current in a series of patients with *SCN5A*-negative BrS, all of whom failed to exhibit structural features of cardiomyopathy.^65^ Functional in vitro evaluation resulted in decreased sodium current at sites of cell-to-cell contact. This was reversed after transfection of wild-type *PKP2* in cellular models but not in mutant forms associated with BrS.

Additional studies have shown deleterious interactions between pathogenic variants in other desmosomal genes and sodium current, as in the cases of *DSG2*,^66^ *DSP*,^67^ and *JUP*.^68^ The clinical phenotype of BrS has also been observed in a patient with a pathogenic *LMNA* variant.^69^ However, such genes currently have insufficient evidence for their inclusion in genetic testing panels for BrS.^70^ Survivors of unexplained cardiac arrest, including patients with iVF, may also harbor pathogenic variants in *SCN5A* in a small proportion, but more significantly, disease-causing variants in cardiomyopathy-related genes, including ACM, have been implicated in 10%.^71^ Indeed, interaction between pathogenic desmosomal variants and calcium current may also represent an important arrhythmia mechanism in the absence of overt structural defects. The concept of a desmosome-dyad axis has been proposed whereby disruption of the desmosome can lead to downregulation of the calcium handling protein integrin β1D, which precipitates hyperphosphorylation of RYR2 (Ser-2030) and predisposes to catecholaminergic polymorphic ventricular tachycardia–like ventricular arrhythmias.^72,73^ Moreover, disruption of calcium current homeostasis has also been reported in *PKP2*-deficient mice, in which an RV-predominant arrhythmogenic substrate was observed in advance of any overt cardiomyopathic changes.^74^ However, we presume that much of the remaining heritability in ERS and iVF could also have a polygenic basis.

## Clinical Overlap With Cardiomyopathy

Over recent years, several studies have reported overlapping clinical features between ACM and BrS in some patients, suggesting that a common disease pathway may underlie such cases.^75,76^ Several investigators have postulated that such changes may relate to changes in the connexome,^77–79^ a network at the intercalated disk that integrates mechanical junctions, gap junctions, and the voltage-gated sodium channel.

**Figure 2.**
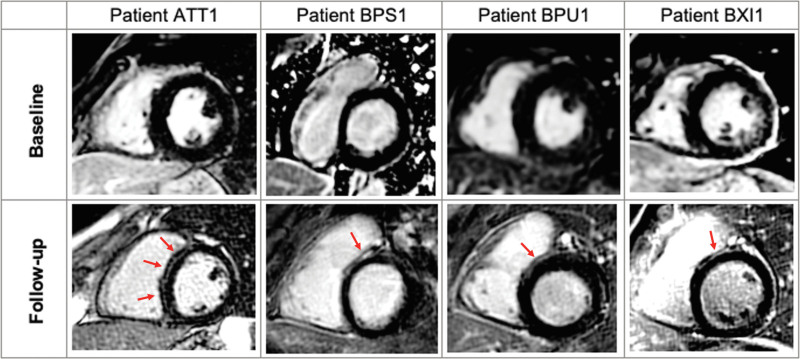
**Development of LGE on CMR imaging during follow-up in patients with BrS.** Four (22%) patients (ATT1, BPS1, BPU1, and BXI1) developed focal septal late gadolinium enhancement (LGE) during assessment with serial cardiac magnetic resonance (CMR). Average time between follow-up imaging was 5.0±1.7 years. BrS indicates Brugada syndrome. Reproduced with permission from Isbister et al.^93^ Copyright © 2023 Elsevier.

**Figure 3.**
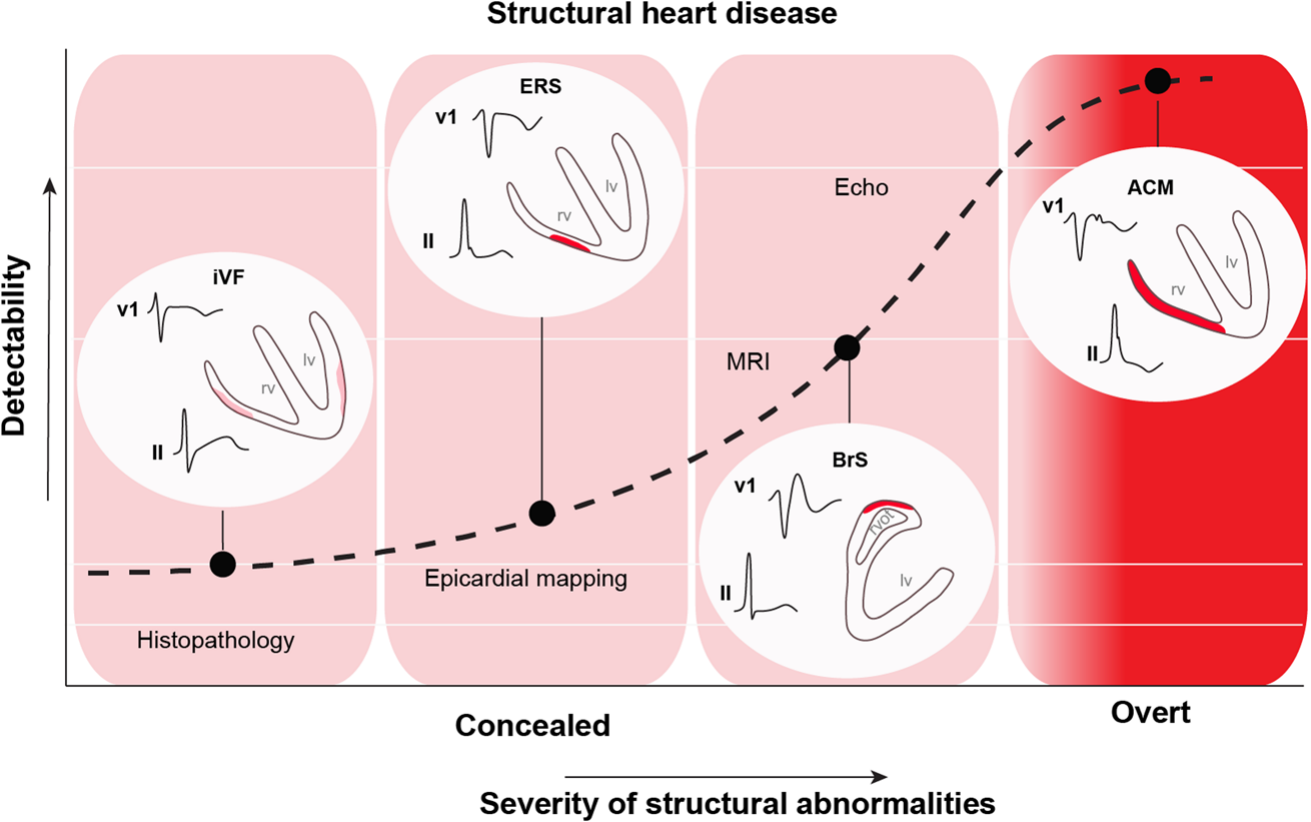
**Detectability and severity of structural abnormalities in BrS, ERS, and ACM with respect to cardiac diagnostic modalities.** ACM indicates arrhythmogenic cardiomyopathy; BrS, Brugada syndrome; ERS, early repolarization syndrome; iVF, idiopathic ventricular fibrillation; and MRI, magnetic resonance imaging. Adapted from Boukens et al^94^ with permission. Copyright © 2020 Elsevier.

Case series have documented RV electromechanical abnormalities (including epsilon waves) in BrS and the presence of a provocable type 1 Brugada ECG in patients meeting task force criteria for ACM.^76,80–82^ Furthermore, the association between BrS and morphological abnormalities of the RV has been explored^83–87^ (Table 1). Gray et al^91^ compared cardiac magnetic resonance (CMR) imaging data among patients with BrS, patients with ACM, and control subjects. The BrS cohort was characterized by increased volumes and abnormal function of the RVOT compared with controls, but, unlike the ACM group, the BrS group did not show global RV dilatation or systolic impairment. Some patients with BrS also demonstrate left ventricular late gadolinium enhancement on CMR,^83^ suggesting a degree of phenotypic overlap with cardiomyopathies such as ACM. One CMR study showed emergence of focal septal late gadolinium enhancement in 4 patients with BrS during follow-up, suggesting that a progressive evolution of imaging abnormalities occurs in some patients (Figure 2).^93^ Despite these reports, not all CMR studies have shown such changes.^90^ Moreover, there are a lack of data indicating evolving myocardial impairment in patients with BrS. This is in line with our view that microstructural changes are minor, undetectable by conventional imaging, and unlikely to cause overt myocardial dysfunction (Figure 3). Nonetheless, in one study, structural RVOT abnormalities appeared to confer a worse prognosis in BrS, representing a potential marker for arrhythmic risk.^95^ The Table 1 summarizes previously published electrophysiological and imaging data on the apparent microstructural substrate present in BrS, ERS, and iVF. To the best of our knowledge, CMR imaging has not been studied in ERS or iVF.

**Table 1.**
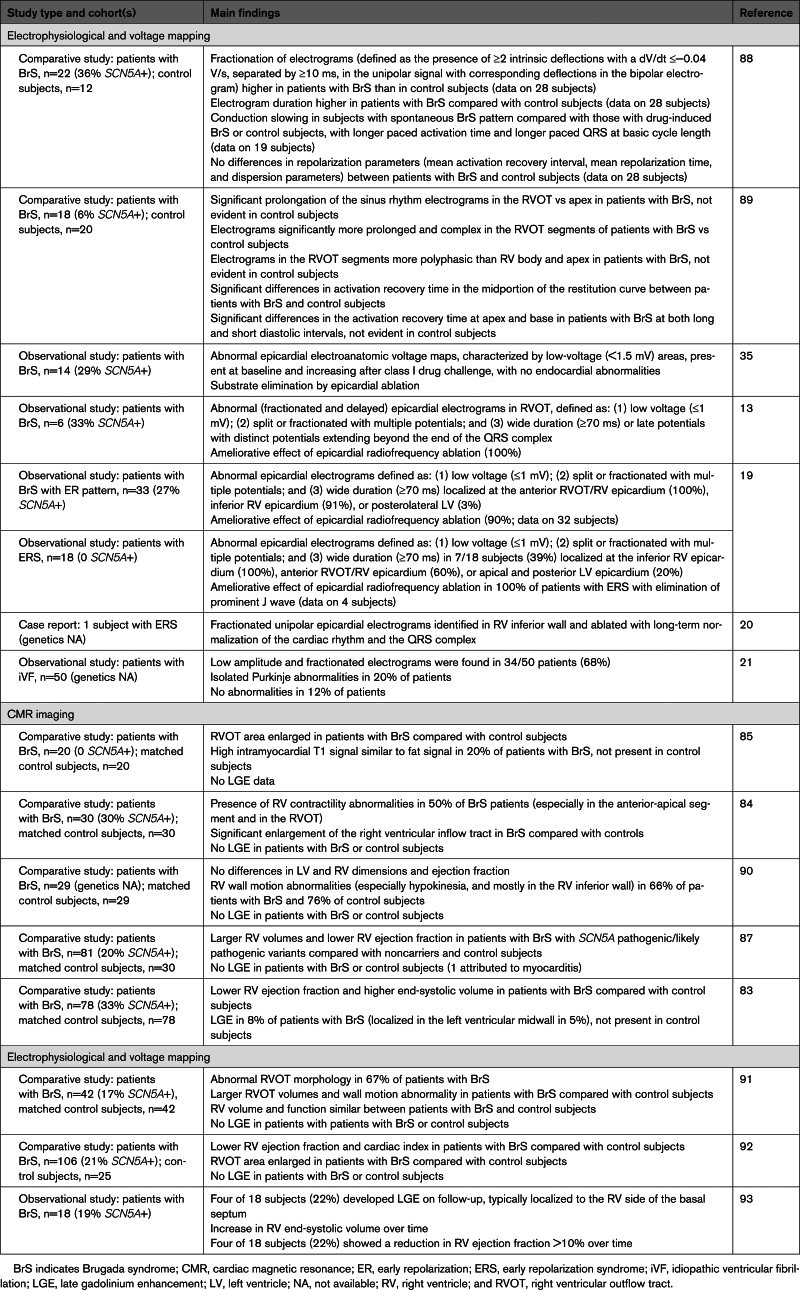
Studies Detailing Electrophysiological and CMR Features of Subjects With BrS, ERS, and iVF

Figure 3 shows the relationship between these disorders and the severity of the microstructural changes with respect to their detectability by various diagnostic methods. Depending on the resolution of the imaging or diagnostic method used, the syndromes are defined as structural heart disease or progressive or not. However, gadolinium enhancement lacks sensitivity for diffuse patterns of interstitial fibrosis, which may be better served by novel imaging techniques such as T1 mapping.

## Predilection of Location

There is a potentially shared predilection of location of the microstructural abnormalities and VF origin in the subepicardial (right) ventricular myocardium in at least a subset of patients with 1 of the 3 syndromes. This raises the question of the cause for this predilection. A possible explanation may be found in cardiac development. The progenitor cells of the left ventricular and RV compartments have a different developmental history and have been exposed to different signals and gene programs before their differentiation.^96^ Studies in chickens have revealed that the RVOT is derived from the outflow tract (OFT) of the embryonic and fetal heart.^97^ The electrophysiological properties and gene expression in the cardiomyocytes of the prenatal OFT differ from those of the ventricles. The prenatal OFT conducts the cardiac impulse slowly, and the main protein responsible for intercellular electrical communication, connexin43, is not expressed in the OFT. Some remnants of these phenotypic differences may be maintained in the OFT, when it matures to form the RVOT. This may explain why the RVOT may form the basis for reentrant arrhythmias that are facilitated by slow conduction and uncoupling.^98^

A role for cardiac development in disease susceptibility is further supported by the genome-wide association study that associated BrS with several common variants in or near genes encoding transcription factors crucial for electrophysiological patterning of the ventricular myocardium during development, such as *TBX5*, *HEY2*, *IRX3*, and *IRX5.*^52^ These transcription factors directly or indirectly modulate the expression of *SCN5A* and could be causally related to reentry by slowing conduction.^50,99,100^ *TBX5* and *IRX3* are expressed predominantly in the ventricular conduction system and have been associated with atrioventricular conduction disturbance and iVF, respectively.^101,102^ *HEY2* and *IRX5* are expressed in the ventricular myocardium and dictate the transmural gradient in *I*_to_.^103,104^ In mice, Irx5 is expressed in an endocardial-to-epicardial gradient and represses the expression of *Kcnd2*, a potassium channel carrying *I*_to_, leading to low *I*_to_ magnitude in the subendocardium. On the other hand, Hey2 is expressed in an epicardial-to-endocardial gradient. Mice heterozygous for Hey2 show reduced *Kcnd2* expression and lower *I*_to_ magnitude in the subepicardium compared with controls, indicating that Hey2 is required for high magnitude of *I*_to_ in the subepicardium.^104^ Computer simulation experiments have shown that large *I*_to_ reduces sodium current, contributes to a slower conduction in the subepicardium than the subendocardium (especially in the presence of sodium channel blockers), and, in the presence of subtle structural discontinuities, facilitates conduction block.^29,105^

## Clinical Implications

Microstructural defects within the cardiac architecture of patients with BrS, ERS, and iVF or their electrophysiological manifestations are increasingly recognized. From our previous hypothesis of impaired epicardial conduction reserve in the RVOT underlying the BrS,^55^ we postulate that impaired conduction, along with genetic and environmental susceptibility, within sites of microstructural discontinuity in patients with BrS and a large proportion of patients with ERS and iVF, precipitates a reduction in epicardial conduction reserve, which, in turn, leads to the arrhythmogenic substrate and can give rise to the electrocardiographic phenotype in BrS and inferolateral J waves in ERS (Figure 1).

There are currently major deficiencies in our ability to diagnose the underlying cause in cases of initially unexplained cardiac arrest with no apparent structural cardiac abnormality. The rapidly expanding use of high-density electroanatomic mapping and digital analysis software may facilitate digital quantification of tissue and cellular components in which histological changes are subtle or localize to particular regions of myocardium. When combined with machine learning algorithms, this may allow artificial intelligence–led diagnostics and a reduced reliance on current qualitative and descriptive techniques used in cardiac pathology. Future studies may also consider mRNA sequencing of tissue specimens to enable transcriptome-wide analysis of molecular pathways implicated in collagen synthesis, which could enable the development of metabolically targeted therapies. Furthermore, modern imaging modalities such as CMR T1 mapping or photon-counting computed tomography have provided an invaluable opportunity to visualize fibrosis patterns in vivo. This could potentially facilitate objective comparisons of such phenotypes and may have implications for clinical practice, particularly early detection of these pathologies. For example, high-resolution imaging techniques used to detect and longitudinally assess myocardial fibrosis could form the basis of future investigations into its role in arrhythmic risk stratification and local therapy. Understanding the histological and electrophysiological substrate may also help in developing morphometric diagnostic criteria for a subepicardial cardiomyopathy.

## Conclusions

BrS, ERS, and iVF potentially form part of a spectrum of a common disease defined by subtle subepicardial microstructural abnormalities: a subepicardial cardiomyopathy. Although genetic susceptibility is uncertain and variable, these microstructural abnormalities are consistent with the electrocardiographic characteristics of each of the syndromes, the mechanism of arrhythmogenesis, and the relationship with modulating genetic and environmental factors.

## Article Information

### Sources of Funding

Dr Miles is the recipient of a British Heart Foundation clinical research training fellowship (FS/18/28/33549). Drs Behr and Miles received research funding from the Robert Lancaster Memorial Fund, sponsored by McColl’s Retail Group Ltd, UK. Dr Wilde receives research funding from CVON (Predict-2). Dr Behr and the GenUCA investigators received research funding from the British Heart Foundation.

### Disclosures

None.
